# Pandemic COVID 19 and healthcare professionals: Mental health impact and depression

**DOI:** 10.1192/j.eurpsy.2023.1706

**Published:** 2023-07-19

**Authors:** R. Ben Soussia, R. Melki, M. H. Aoun, W. Bouali, L. Zarrouk

**Affiliations:** Department of psychiatry, Hopital Universitaire Taher SFAR Mahdia, Mahdia, Tunisia

## Abstract

**Introduction:**

The SARS-COV2 pandemic represents a problematic and disruption of global health. Repeated exposure to stressful situations leads to increased psychological distress.

**Objectives:**

To determine the psychoaffective impact of the Covid-19 pandemic on the mental health of health professionals in Tunisia, assess the intensity of depressive symptoms professionals and determine factors associated with the development of these symptoms.

**Methods:**

This was a multicenter, cross-sectional, descriptive and analytical study conducted among health professionals, from May 2^nd^, 2020 to June 30^th^, 2020 in Tunisia. The health professionals included were physicians, nurses, dentists, and pharmacists. Using an electronic form << Google Form >>, a questionnaire was drawn up with 32 items. Assessment of depressive symptoms was performed using the PHQ-9 psychometric scale.

Statistical analysis was performed using SPSS 21.0 statistical software. Regarding the analytical study, a professional with a PHQ-9 scale score between 10 and 27 was considered as having moderate to severe depressive symptoms while a score between 15-27 was in favor of severe depressive symptoms.

**Results:**

Caregivers were predominantly female 69.5% with a mean age of 30.74 years. Anxiety-depressive psychiatric history was found in 11.8% of the subjects. The majority of the professionals were doctors (77.8%) and 9.4% of the participants were nurses. The majority of participants worked in university hospitals (84.2%). One third of the participants, (34.3%) worked in departments with Covid-19 patients with respective rates of 57% for nurses and 36% for physicians. Dentists and pharmacists did not work in Covid-19 circuits.

The mean score on the PHQ-9 scale was equal to 8.62 ± 5.35. Depressive symptoms were noted in 37.4% of the professionals, with moderate to severe intensity in 35.5% of cases. Participants with a psychiatric history of depression or anxiety disorder had significantly higher depressive symptom scores (p<0.001) with 6 times higher the risk of developing moderate to severe depressive symptoms (p<0.0001, OR 6.25, CI [2.35-16.61] and almost 3 times higher the risk of experiencing severe depressive symptoms (p=0.05, OR=2.93, CI [1.09-7.88]). The nursing profession had high odds ratios for the occurrence of moderate to severe depressive symptoms (p=0.002, OR=4.41, CI [1.58-12.28]) and severe depressive symptoms (p=0.02, OR=3.82, CI [1.28-11.39]). A significant relationship was established, between the development of depressive symptoms of moderate to severe intensity with the history of depressive disorder or anxiety disorder (p=0.001) and the nursing profession (p=0.01).

**Conclusions:**

The optimization of prevention, the creation of specific treatment, the promotion of health education and specific hygiene rules would participate in improving the mental health of health professionals.

**Disclosure of Interest:**

None Declared

